# Psychedelics and psychiatric disorders: A emerging role

**DOI:** 10.1192/j.eurpsy.2021.2054

**Published:** 2021-08-13

**Authors:** C. Peixoto, F. Queirós Santos, D. Rego, H. Medeiros

**Affiliations:** 1 Psychiatry, Hospital do Divino Espírito Santo de Ponta Delgada, E.P.E., Ponta Delgada, Ponta Delgada, Portugal; 2 Psychiatry, Centro Hospitalar Universitário do Algarve, Faro, Portugal

**Keywords:** Psilocybin, MDMA, psychedelics, LSD

## Abstract

**Introduction:**

Recently there has been renewal in interest of psychedelic research. Classic psychedelics such as lysergic acid diethylamide (LSD), psilocybin and mescaline act pharmacologically as agonists at the 5-HT2A receptor. The entactogens like methylenedioxymethamphetamine (MDMA), acts as a serotonin, dopamine and noradrenaline agonist. All of these drugs are potential candidates in the treatment of multiple psychiatric illnesses.

**Objectives:**

The authors intend to review the literature on the clinical application of psychedelic drugs in psychiatric disorders.

**Methods:**

Non-systematic review of the literature.

**Results:**

In recent clinical trial the psychedelic is given with psychotherapeutic input. In a supportive setting, psychedelics produced immediate and significant anti-depressant and anxiolytic effects that were endured for several months. Randomized clinical trials support the efficace of psilocybin in the treatment of depression and those with anxiety and depression symptoms provoked by life-threatening cancer. There have also been studies showing efficacy in both alcohol and tobacco dependence. When administered safely LSD can reduce anxiety and have anti-addictive property. Randomized clinical trials support the efficacy of MDMA in the treatment of PTSD. Psychedelics were well-tolerated, few adverse effects have been reported. The most common adverse effects were transient anxiety, short-lived headaches, nausea and mild increases in heart rate and blood pressure, with no persisting adverse effects. Serious adverse events, such as persistent psychosis and suicidality, have not been demonstrated.

**Conclusions:**

Psychedelics appear to be effective in multiple psychiatric disorders and are well-tolerated, although further evidence is required, to better see they therapeutic potential.

**Disclosure:**

No significant relationships.

